# The Liver the Seat of Formation of Urea

**Published:** 1871-02

**Authors:** 


### The Liver the Seat of Formation of Urea.

The latest researches upon the place of formation of urea, and especially the beautiful experiments of M. Grehant, have demonstrated that the kidneys are by no means secretory, but purely excretory organs for urea. Dr. Cyon, in the last number of the *Centralblatt*, publishes a few facts in the form of a provisional communication, to show that it is probably produced at the liver. The plan of experimentation adopted, in common with M. Istomin, was as follows: The whole of the blood was abstracted from the carotid of a dog, and a portion, after being defibrinated, was transmitted, by means of mercurial pressure, through the liver. Coincidently three canulæ were introduced—one into the inferior vena cava, the second into the hepatic artery, and the third into the vena porta.. The results of careful analysis showed that the blood which had passed through the liver contained a much larger proportion of urea than ordinary arterial blood. In one experiment 100 c. c. of the arterial blood, when defibrinated, contained 0.08 grammes of urea; but after having been passed four times through the liver, the same quantity contained 0.176 grammes.—*Medical Record.*

